# Role of nutrition support in adult cardiac surgery: a consensus statement from an International Multidisciplinary Expert Group on Nutrition in Cardiac Surgery

**DOI:** 10.1186/s13054-017-1690-5

**Published:** 2017-06-05

**Authors:** Christian Stoppe, Andreas Goetzenich, Glenn Whitman, Rika Ohkuma, Trish Brown, Roupen Hatzakorzian, Arnold Kristof, Patrick Meybohm, Jefferey Mechanick, Adam Evans, Daniel Yeh, Bernard McDonald, Michael Chourdakis, Philip Jones, Richard Barton, Ravi Tripathi, Gunnar Elke, Oliver Liakopoulos, Ravi Agarwala, Vladimir Lomivorotov, Ekaterina Nesterova, Gernot Marx, Carina Benstoem, Margot Lemieux, Daren K. Heyland

**Affiliations:** 1 Department of Intensive Care Medicine, University Hospital of the RWTH Aachen, Pauwelsstraße 30, 52074 Aachen, Germany; 2 Department of Thoracic, Cardiac and Vascular Surgery, University Hospital, RWTH Aachen, Pauwelsstraße 30, 52074 Aachen, Germany; 3 Cardiac Surgical Intensive Care, Johns Hopkins Hospital Baltimore, Blalock 618, 600 N. Wolfe Street, Baltimore, MD 21287 USA; 4 Department of Anesthesia, Royal Victoria Hospital, McGill University Health Centre, Montreal, Canada; 5 Department of Microbiology and Immunology, McGill University Health Centre, Montreal, Canada; 6 Department of Anaesthesiology, Intensive Care Medicine and Pain Therapy, University Hospital Frankfurt, Theodor-Stern-Kai 7, 60590 Frankfurt am Main, Germany; 7 Division of Endocrinology, Diabetes, and Bone Disease, Icahn School of Medicine at Mount Sinai, New York, NY USA; 8 Icahn School of Medicine at Mount Sinai, New York, NY USA; 9 Department of Surgery, Division of Trauma, Emergency Surgery and Surgical Critical Care, Massachusetts General Hospital, Boston, MA USA; 10 Division of Cardiac Anesthesiology and Critical Care Medicine, University of Ottawa Heart Institute, Ruskin Street H2410, Ottawa, ON K1Y 4W7 Canada; 11 Department of Medicine, Aristotle University of Thessaloniki, University Campus, Thessaloniki, 54124 Greece; 12 Departments of Anesthesia & Perioperative Medicine and Epidemiology & Biostatistics, University of Western Ontario, London, Canada; 13 Department of Surgery, University of Utah School of Medicine, Salt Lake City, UT USA; 14 Department of Anesthesiology, The Ohio State University College of Medicine, Columbus, OH USA; 15 Department of Anesthesiology and Intensive Care Medicine, University Medical Center Schleswig-Holstein, Campus Kiel, Arnold-Heller-Str. 3 Haus 12, 24105 Kiel, Germany; 16 Department of Cardiothoracic Surgery, Heart Center, University of Cologne, Cologne, Germany; 17 Department of Anesthesiology, Section on Critical Care Medicine, Wake Forest School of Medicine, Medical Center Boulevard, Winston-Salem, NC 27157 USA; 18 Department of Anesthesiology and Intensive Care, Research Institute of Circulation Pathology, Novosibirsk, Russia; 19 Department of Anesthesiology and Intensive Care Medicine, National Pirogov Surgical Medical Center, Moscow, Russia; 20 Department of Critical Care Medicine, Queen’s University and Clinical Evaluation Research Unit, Angada 4, Kingston General Hospital, Kingston, ON K7L 2V7 Canada

**Keywords:** High-risk cardiac surgery, Cardiopulmonary bypass, Systemic inflammatory response, Organ dysfunctions, Nutrition risk stratification, Underfeeding, Postoperative nutritional management, Supplemental parenteral nutrition, Enteral nutrition, Pharmaco-nutrition

## Abstract

Nutrition support is a necessary therapy for critically ill cardiac surgery patients. However, conclusive evidence for this population, consisting of well-conducted clinical trials is lacking. To clarify optimal strategies to improve outcomes, an international multidisciplinary group of 25 experts from different clinical specialties from Germany, Canada, Greece, USA and Russia discussed potential approaches to identify patients who may benefit from nutrition support, when best to initiate nutrition support, and the potential use of pharmaco-nutrition to modulate the inflammatory response to cardiopulmonary bypass. Despite conspicuous knowledge and evidence gaps, a rational nutritional support therapy is presented to benefit patients undergoing cardiac surgery.

## The scientific rationale for nutrition therapy in the cardiac surgery ICU

Patients undergoing cardiac surgery are routinely exposed to significant systemic inflammation, causing organ injury and dysfunction. Cardiopulmonary bypass (CPB) triggers systemic inflammatory response syndrome (SIRS) with release of reactive oxygen species (ROS) and reactive nitrogen species (RNS) and mainly pro-inflammatory cytokines [1–3]. This often results in serious life-threatening complications with loss of physical capacity, associated with prolonged critical illness, which may negate any benefit from correction of the original, underlying cardiac disease. Such patients require aggressive, life-sustaining therapies to promote organ recovery and mid to long-term outcome advantages [4].

Underfeeding is a major issue in cardiac surgery patients [5, 6]. Table 1 demonstrates an overview of clinical studies investigating nutritional support in patients undergoing cardiac surgery. No sufficiently designed, adequately powered, randomized controlled trials investigating the effect of nutritional therapy initiated early in high-risk cardiac patients after surgery are available. Yet, several small studies have provided initial evidence on the feasibility and clinical significance of nutritional therapy in cardiac surgery patients. We therefore aimed to develop an expert-derived consensus on best nutritional practices in this patient population.

**Table 1:** Overview of studies investigating nutritional protocols in cardiac surgical patients

Reference	Study design	Population	Off-pump or on-pump procedure	Intervention/comparison	Summary of results
Rahman 2016 [48]	POS	Patients (n = 787) after cardiac surgery	No information	60.1% of patients received artificial nutrition support. Of these patients, 78% received enteral nutrition (EN) alone, 17% received a combination of EN and parenteral nutrition (PN), and 5% received PN alone. The remaining 314 patients (40%) received no nutrition	The mean (SD) time from ICU admission to EN initiation was 2.3 (1.8) days. The adequacy of calories was 32.4% ± 31.9% from EN and PN and 25.5% ± 27.9% for patients receiving only EN. In EN patients, 57% received promotility agents and 20% received small bowel feeding. There was no significant relationship between increased energy or protein provision and 60-day mortality. Postoperative cardiac surgery patients who stay in the ICU for 3 or more days are at high risk for inadequate nutrition therapy
Flordelis Lasierra 2015 [49]	POS	Cardiac surgery patients (n = 37) with hemodynamic failure requiring more than 24 h of mechanical ventilation	No information	Early EN protocol of institution	11 patients (29.7%) required mechanical circulatory support, and 25 (68.0%) met the criteria for early multiorgan dysfunction. Mortality was 13.5%. Mean EN duration was 12.3 days (95% confidence interval (CI) 9.6–15.0). The mean EN diet volume delivered/patient/day was 1199 mL (95% CI 1118.7–1278.8), and mean EN energy delivered/patient/day was 1228.4 kcal (95% CI 1145.8–1311). The set energy target was achieved in 15 patients (40.4%). The most common EN-related complication was constipation. No case of mesenteric ischemia was detected. Early EN is feasible in cardiac surgery patients and not associated with serious complications, but appropriate energy target by EN alone cannot be attained
Visser 2014 [50]	RCT	Patients (n = 33) undergoing off-pump coronary artery bypass grafting	Off-pump	Enteral, parenteral, or no nutrition (control) from 2 d before, during, and until 2 d after surgery	The myocardial arginine:ADMA ratio increased during surgery and was significantly higher in the enteral and parenteral groups than in the control group (median (IQR) 115.0 (98.0–142.2) (*P* = 0.012), 116.9 (100.3–135.3) (*P* = 0.004), and 93.3 (82.7–101.1)). The change in the preoperative to postoperative plasma arginine:ADMA ratio correlated with the change in myocardial glucose metabolism in positron emission tomography (*r* = 0.427, *P* = 0.033). Enteral or parenteral nutrition before, during, and after CABG may positively influence myocardial glucose metabolism by increasing the plasma and myocardial arginine:ADMA ratio
Umezawa Makikado 2013 [51]	POS	Cardiac surgery adult patients (n = 7) receiving VA ECMO for severe hemodynamic failure unresponsive to conventional therapies	No information	ICU EN Protocol (25 kcal/kg, to be reached over 4 days)	Two patients received EN within the first 24 h, and all patients were on EN by 48 h. More than 70% nutritional therapy (NT) was achieved within the first week in all cases. After 2 weeks, only 3 of 7 patients continued on ECMO, and a decrease in NT was noted. By day 7, the mean energy balance was –245.99 (range –75.82 to –555) kcal/day and was –373 (range –75.82 to –795) kcal/day at the end of the study. Mean cumulated deficit by day 7 was –603.54 (range –75.82 to –1721.92) kcal/day and was –3264.94 (range –75.82 to –7087.08) kcal/day at the end of the study. No serious adverse events were attributable to EN, such as aspiration of gastric contents, abdominal distension, ileus, vomiting, gastrointestinal bleeding, or bowel ischemia. Four patients met the criteria for constipation. Ventilator-associated pneumonia was diagnosed in one patient. One patient presented with a catheter-associated bloodstream infection Early EN is possible and safe in patients with severe hemodynamic failure receiving VA ECMO.
Tepaske 2007 [42]	RCT	Patients (n = 49) scheduled to undergo cardiac surgery with the use of extracorporeal circulation, aged 70 years or older, had a compromised left ventricular function, or were planned for mitral valve surgery	On-pump	Addition of glycine to a standard preoperative oral immune-enhancing nutrition supplement (OIENS)	Infectious morbidity was significantly lower in both treatment groups compared with the control group (*P* = 0.02). Less supportive therapy was necessary to stabilize circulation in both treatment groups compared with the control group. Median length of hospital stay was 7.0, 6.5, and 8.0 days in the OIENS + glyc, OIENS, and control groups, respectively. Inflammatory responses, as measured by systemic levels of pro-inflammatory cytokines and surface markers on polymorphonuclear cells were comparable for all groups. Preoperative OIENS reduces postoperative infectious morbidity and results in more stable circulation; the addition of glycine does not result in any beneficial effect over standard OIENS
Rapp-Kesek 2007 [52]	RCT	Patients (n = 16) undergoing cardiac surgery	No information	Enteral nutrition and dopexamine	Dopexamine and enteral nutrition caused no adverse effects on oxygen consumption or the oxygen extraction ratio. Enteral nutrition did not increase the splanchnic blood flow or cardiac index. Dopexamine increased the systemic blood flow with only a transient effect on the splanchnic blood flow. Dopexamine increased the lactate concentration, possibly indicating a more ischemic condition.
Berger 2005 [53]	POS	Patients (n = 70) after cardiac surgery with extracorporeal circulation, staying 5 days in the ICU, with acute cardiovascular failure	On-pump	Enteral nutrition	Forty patients required artificial nutrition. Energy delivery was very variable. There was no abdominal complication related to EN. As a mean, 1360+/-620 kcal/kg/day could be delivered enterally during the first 2 weeks, corresponding to 70+/-35% of energy target. Enteral nutrient delivery was negatively influenced by increasing dopamine and norepinephrine doses, but not by the use of IABP. EN is possible in the majority of patients with severe hemodynamic failure, but usually results in hypocaloric feeding. EN should be considered in patients with careful abdominal and energy monitoring
Sustić 2005 [54]	RCT	Patients (n = 40) after coronary artery bypass grafting	No information	Early EN and effects of metoclopramide	Enteral feeding with isoosmotic enteral formula was initiated by nasogastric tube 18 h after surgery. After 6 h, feeding was stopped, and paracetamol solution (1000 mg) and 10 mg of metoclopramide IV or 2 ml of saline IV were concurrently administered. The plasma paracetamol concentrations 15, 30, 60, and 120 minutes after the administration of paracetamol were significantly higher in metoclopramide group than in control group: (*t*(+15)) 5.4-/+2.7 vs 3.3-/+2.5 (Mann-Whitney *U* test; *P* = 0.017); (*t*(+30)) 6.7-/+2.4 vs 3.7-/+2.0 (*P* = 0.006); (*t*(+60)) 7.7-/+2.5 vs 5.1-/+3.2 (*P* = 0.008); (*t*(+120)) 8.5-/+2.2 vs 5.2-/+2.8 (*P* = 0.005). The AUC value was 34% larger in the metoclopramide group vs control group (574-/+296 vs 429-/+309; *P* = 0.027). There were no significant differences in gallbladder ejection fraction between groups (group metoclopramide vs control group: (*t*(0)-t(+15)) –2% vs –2%; (*t*(+15)-t(+30)) 1% vs 4%; (*t*(+30)-*t*(+60)) 0% vs –1%; (*t*(+60)-*t*(+120)) 1% vs 3%; *P* = NS). In CABG surgery patients with early enteral feeding, a single dose of intravenous metoclopramide effectively improves gastric emptying, but does not have any prokinetic effect on gallbladder motility
Hartwell 2003 [55]	POS	Patients (n = 15) after coronary artery bypass grafting	No information	Dietary advice	Dietary intake was assessed on three occasions (preoperatively, 2 months after surgery and 1 year after surgery) by use of a food amount frequency questionnaire that had been previously validated. Patients were also asked to provide information on any dietary advice they had received. The absolute mean intakes of total fat, saturated fat and dietary cholesterol significantly increased 1 year after CABG surgery by 21%, 36% and 51%, respectively, and the choice of food items reflected this change in nutrient intake. These undesirable changes occurred despite the provision of dietary advice.
Kesek 2002 [56]	POS	Patients (n = 73) scheduled for coronary artery bypass grafting and/or valvular disease, thoracic or thoraco-abdominal aortic aneurysms or other combined procedures	No information	Enteral nutrition	In 59/73 patients, EN was started within 3 days. EN was discontinued in half of the patients when they were able to feed themselves. Twelve patients vomited, one of them severely. Dislocation of the nasogastric tube occurred in 28 patients. The 15 patients with diarrhoea were treated with 2–6 broad-spectrum antibiotics during their ICU stay. Out of 73 patients, 40 did not show any gastric residual volume (GRV). GRV decreased during EN in 50% of the patients with fairly large or large residual volumes. The incidence of aspiration pneumonia was 10%. In the cardiothoracic ICU, individually adjusted early EN is feasible with few problems
Tepeske 2001 [57]	RCT	Patients (n = 50) scheduled for coronary artery bypass grafting	No information	Oral immune-enhancing nutritional supplementation containing L-arginine, omega3 polyunsaturated fatty acids, and yeast RNA (n = 25), or a control (n = 25) for a minimum of 5 days	Preoperative expression of HLA-DR epitopes on monocytes was significantly higher in patients given the study treatment (109% (95% CI 92–128)) than those given the control (69% (58–82)) compared with baseline (100%) (*P* = 0.02, repeated measures ANOVA). However, concentration of interleukin 6 was significantly lower in the treatment group (0.90 pg/L (0.69–1.18)) than in the control group (1.94 pg/L (1.45–2.59)) (*P* = 0.032, repeated measures ANOVA). Additionally, delayed-type hypersensitivity response to recall antigens improved preoperatively and remained better until hospital discharge
Revelly 2001 [58]	POS	Patients (n = 9) 1 day after cardiac surgery under cardiopulmonary bypass	On-pump	Isoenergetic EN via a post pyloric tube while catecholamine treatment remained constant. Baseline (fasted) condition was compared to continuous EN condition	Cardiac index (CI), mean arterial pressure (MAP), pulmonary and wedge pressures, indocyanine green (ICG) clearance, gastric tonometry, plasma glucose and insulin, and glucose turnover (6,62H2-glucose infusion) were determined repetitively every 60 minutes during 2 h of baseline fasting condition and 3 h of EN. During EN, CI increased (from 2.9 +/- 0.5 to 3.3 +/- 0.5 l min-1 m-2), MAP decreased transiently (from 78 +/- 7 to 70 +/- 11 mmHg), ICG clearance increased (from 527 +/- 396 to 690 +/- 548 ml/min), and gastric tonometry remained unchanged, while there were increases in glucose (158 +/- 23 to 216 +/- 62 mg/dl), insulin (29 +/- 23 to 181 +/- 200 mU/L), and glucose rate of appearance (2.4 +/- 0.2 to 3.3 +/- 0.2 mg min-1 kg-1). The introduction of EN in these postoperative patients increased CI and splanchnic blood flow, while the metabolic response indicated that nutrients were utilized. These preliminary results suggest that the hemodynamic response to early EN may be adequate after cardiac surgery even in patients requiring inotropes
Berger 2000 [28]	POS	Cardiac patients (n = 39)	No information	Early EN in patients with adequate hemodynamic status, and in patients with hemodynamic failure	Absorption was strongly reduced on day 1 in all patients after gastric administration (lower peak paracetamol and AUC), but normal after post pyloric delivery. Duration of anesthesia and of circulatory bypass did not affect paracetamol absorption. On day 3, AUC was close to normal in the case of hemodynamic failure. Peak absorption on day 1 was negatively correlated with opiate dose (*r*2 = 0.176, *P* = .008). Hypocaloric enteral nutrition was well-tolerated
Fiaccadori 1994 [59]	POS	Cardiac patients undergoing mitral valve replacement (n = 12)	On-pump	A lipid emulsion containing 10% medium-chain triglycerides (MCT) and 10% long-chain triglycerides (LCT) was infused at a rate of 1 ml/kg/h (3.3 mg/kg/min) for 2 h to 24 h after surgery	Fat emulsions containing both MCT and LCT, when given at 3.3 mg/kg/min for 120 minutes following valvular heart surgery, do not exert negative cardiopulmonary effects and could represent a source of rapidly metabolized substrates
Behrendt 1984 [60]	RCT	Cardiac patients undergoing CABG (n = 40)	No information	Patients were treated with 3 types of hypocaloric parenteral nutrition consisting of the same content of amino acids (75 g/day) but different amounts of carbohydrates: 120 g dextrose, 120 or 200 g dextrose/fructose/xylitol (1:2:1) per day; 18 patients received only 75 g/day of dextrose (controls)	No beneficial effects on the concentrations of visceral serum proteins (transferrin, pre-albumin, retinol binding protein, cholinesterases) were found
Weidler 1984 [61]	RCT	Cardiac patients (n = 20)	No information	Both groups received identical carbohydrate calories (2000 kcal/day) group 1 received only essential amino acids while in group 2 a combined pattern of essential and non-essential amino acids were infused	Routine parameters evaluating protein metabolism in both groups did not differ significantly. Nitrogen balance was positive only in group 2; the difference between the two groups concerning other parameters was minimal
Haider 1981 [62]	RCT	Patients (n = 22) after open heart surgery	On-pump	Control group received Ringer lactate as postoperative infusion (RL-group), the 2nd group was given 50% glucose (0.5 g/kg/h) and insulin (250 U/1000 cc) (GI-group)	In contrast with the RL-group in the GI-group there was a significant decrease in FFA-serum level and cAMP serum level, which developed during the infusion. Urine output and urinary glucose excretion was nearly equal in both groups. Urinary potassium excretion in the GI-group remained significantly one third lower than that of the RL-group, in spite of the potassium supply to the GI-group being nearly twice as much and serum potassium level approximately equal. Urinary sodium excretion in the GI-group on the other hand was approximately 15% higher than that in the RL-group. In relation to preoperative values, postoperative urinary N-excretion in the GI-group was unchanged, whereas in the RL-group the postoperative N-excretion was significantly 30% increased; in postoperative alpha-amino-N-excretion there was only a small difference between the groups, which indicates an insulin-modifiable increase in protein breakdown rather than decreased protein synthesis
Fisch 1981 [63]	RCT	Patients (n = 13) following cardiopulmonary bypass procedures	No information	Two intravenous fat emulsions (Liposyn 10%, Abbott Laboratories, North Chicago, IL, USA and Intralipid 10%, Cutter Laboratories, Berkeley, CA, USA)	Neither intravenous fat emulsion was observed to exert significant changes in left ventricular stroke work, left ventricular filling pressure, cardiac output, systemic vascular resistance, mean systemic arterial blood pressure, central venous pressure, or mean pulmonary artery pressure. This study confirmed that the administration of 10% fat emulsions available in the USA does not exert significant untoward hemodynamic changes, even in patients with severe cardiac impairment recovering from recent open-heart surgery
Abel 1976 [64]	RCT	Malnourished (n = 44) and non-malnourished adult patients (n = 20) after cardiac surgery	No information	Parenteral nutrition compared to routine postoperative infusions for 5 days or until oral intake was deemed adequate	The overall mortality in the malnourished group was 12.5%, in the malnourished patients receiving parenteral nutrition it was 20% and in the non-malnourished group it was 0%. Malnutrition itself appears to significantly increase morbidity and mortality following cardiac surgery. Although total parenteral nutrition can safely be administered in the early postoperative period following open heart surgery, an average of 5 days of prophylactic parenteral nutrition did not appear to be effective in lessening the untoward effects of the malnourished state

Studies were included if they met the following criteria: (1) population: critical ill, adult patients after cardiac surgery; (2) intervention and comparison: any type of nutritional protocol, e.g., early aggressive vs early lower-dose enteral nutrition, early vs delayed enteral nutrition, enteral nutrition alone versus enteral nutrition plus supplemental parenteral nutrition; (3) outcome: mortality (ICU, hospital, long-term), length of stay (ICU, hospital), infectious and non-infectious complications, quality of life, physical rehabilitation; (4) type of studies: prospective or retrospective observational studies, randomized or non-randomized clinical trials, systematic reviews of literature and meta-analyses. Medical Subject Headings (MeSH) is the United States National Library of Medicine (NLM) comprehensive controlled vocabulary thesaurus for the purpose of indexing journal citations with concepts from all domains of the biomedical domain. It was applied to identify the most suitable keywords and search terms. Consequently, the following search strategy (example given for MEDLINE) was developed to identify matching studies: ((Nutrition Therapy[MeSH Major Topic]) AND (cardiac[Title/Abstract] OR heart[Title/Abstract])) AND surg*[Title/Abstract]. To locate relevant articles, six bibliographic databases (Cochrane Database of Systematic Reviews (CDSR), Cochrane Central Register of Controlled Trials (CENTRAL), Database of Abstracts of Reviews of Effects (DARES), and the Medical Literature Analysis and Retrieval System Online (MEDLINE) were searched on 1 November 2016. In addition, reference lists of matching studies, personal files, and relevant review articles were searched for additional studies. No time or language restrictions were applied, if at least the abstract was available in English or German. *Abbreviations*: *POS* prospective observational study, *RCT* randomized controlled trial, *EN* enteral nutrition, *PN* parental nutrition, *CABG*, coronary artery bypass grafting, *ECMO*, extracorporeal membrane oxygenation, *NT* nutritional therapy, *VA* ventilator assist, *glyc* glycine, *OIENS* oral immune-enhancing nutrition supplement, *IAB*P intra-aortic balloon pump, *AUC* area under the curve, *NS* not significant, *ANOVA* analysis of variance, *CI* cardiac index, *MAP* mean arterial pressure, *ICG* indocyanine green, *MCT* medium-chain trigylcerides, *LCT* long-chain triglycerides, *FFA* free fatty acids

## Nutrition in cardiac surgery patients

Preoperative fasting sets the stage for catabolic stress [7], insulin resistance [8], nutrient deficiencies, and adverse immune function [9]. During cardiac surgery, patients commonly receive only intravenous crystalloid solutions, which are continued for several days postoperatively [9].

Considering the postoperative course, in a retrospective analysis of about 5400 mechanically ventilated patients, cardiac surgery was most associated with iatrogenic malnutrition [5]. This alarming finding is compounded by observations that nutrition support was implemented later and with the lowest nutritional adequacy in the cardiac surgery population compared to all other surgical or medical ICU patients [5]. Recently, Rahman et al. [6] evaluated nutrition practices in cardiac surgery patients and demonstrated that nutrition support was insufficient with respect to energy and protein needs. Patients only received approximately 50% of what was prescribed. Importantly, an improvement in 60-day mortality with greater nutrition intake could not be demonstrated. This observation raises the question whether all cardiac surgery patients benefit the same from artificial nutrition therapy or whether there are specific subgroups of cardiac surgery patients that will benefit more.

As society ages, older patients are presenting for cardiac surgery with an increased prevalence of comorbidities. In addition, the number of patients with advanced heart failure has increased and the implantation of pulsatile-flow ventricular assist devices (VAD) has become an established therapeutic strategy to improve survival rates and quality of life [10]. Malnutrition may be a significant comorbidity and driver for dysfunction of many organ systems. This can exacerbate an already impaired organ reserve, increasing susceptibility to operative trauma, ischemia/reperfusion injury, anesthesia-related complications, and inflammation. Cardiac patients who are well-nourished prior to surgery experience less morbidity and mortality than those who are malnourished [11, 12]. Several observational studies have noted the importance of energy and protein metabolism in the early recovery period after cardiac surgery, documenting significant postoperative depletion of macronutrients and micronutrients [11–14]. Adequate nutritional therapy was suggested to improve patients’ outcomes through maintenance of energy metabolism, gut integrity, microbial diversity and improved wound healing [15]. In summary, preoperative nutritional status and postoperative nutritional management may represent important drivers for clinical outcomes in patients undergoing cardiac surgery, who are at high nutritional risk, which will be discussed in the following section.

**Fig. 1:**
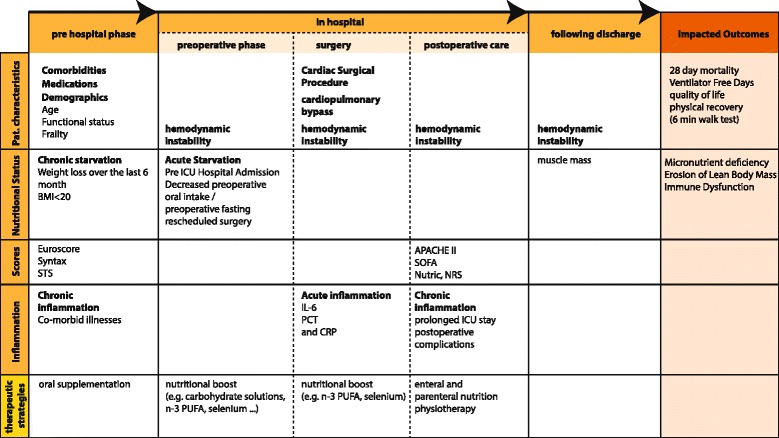
Organizing framework malnutrition and undernutrition and impact on outcomes in cardiac surgery patients. The patients’ preoperative, intraoperative and postoperative time windows comprise specific variables, which may be of particular relevance for potential nutrition support and patients’ outcomes after surgery. Notably, additional studies are encouraged to identify further relevant factors and to validate its clinical significance. *STS* Society of Thoracic Surgeons, *BMI* body mass index, *ICU* intensive care unit, *APACHE II* Acute Physiology And Chronic Health Evaluation II, *SOFA* sequential organ failure assessment, *Nutric* Nutrition Risk in the Critically ill, *CRP* C reactive protein, *IL* interleukin, *PCT* procalcitonin, *PUFA* polyunsaturated fatty acids, *SYNTAX* scoring system to guide decision between coronary artery bypass graft (*CABG*) surgery or percutaneous intervention (*PCI*), *NRS* nutritional risk score

Figure 1 demonstrates selected key factors, which are considered to crucially influence the nutritional state and potential need for intense nutrition therapy in cardiac surgery patients.

## Nutrition risk stratification in cardiac surgery patients

Selection of patients who will benefit most from nutrition support in the postoperative period is critical, but has not been established or standardized. When developed, this selection process would be based on a combination of clinical and biochemical parameters related to validated risk scores for nutrition, cardiac surgery, critical illness, and emerging markers of systemic inflammation, particularly those related to cardiopulmonary bypass and postoperative ICU pharmacology and technology.

### Preoperative nutrition risk assessment

Several scores or assessment tools have been introduced to enable the quantification of nutrition risk. These tools were neither developed for nor validated in critically ill patients [16]. Therefore, the measurement of a patient’s current nutritional status predominantly identifies those who *have already* reached an undernourished general state. *To foresee* an aggravation in the nutritional status, an assessment of nutritional risk must also identify patients at a pre-critical level of malnutrition, who will benefit (and not be harmed) by nutrition support. The Malnutrition Universal Screening Tool (MUST), the Mini Nutritional Assessment (MNA), the Short Nutritional Assessment Questionnaire (SNAQ), the Malnutrition Screening Tool (MST), and the Subjective Global Assessment (SGA) [16] are well-established assessment tools used to evaluate nutrition status in clinical practice.

Lomivorotov and colleagues demonstrated that in patients undergoing cardiac surgery, detection of malnutrition is associated with prolonged ICU stay (>2 days) and both MUST and MNA have independent predictive accuracy with regard to postoperative complications [14]. In a subsequent study, the authors further detected that the SNAQ and MUST have comparable accuracy in detecting malnutrition. Nevertheless, the authors acknowledge that whether preoperative nutritional therapy would improve the outcome in malnourished patients needs to be studied [13]. In fact, no validated pre-surgical scoring system is available to identify patients at an early enough time point for preoperative nutritional replenishment.

### Postoperative nutrition risk assessment to identify patients who may benefit from nutrition therapy

As the aforementioned tools consider all critically ill patients at high risk for malnutrition, the Nutrition Risk in the Critically ill (NUTRIC) score was developed to define nutrition risk in the critically ill ICU patients [17–19]. The observation that not all ICU patients will respond the same to nutritional interventions was the critical driver for the development of the NUTRIC score [17, 20]. Yet, the NUTRIC score has not yet been validated in cardiac surgery patients.

Considering the critically ill, the Nutritional Risk Score (NRS)-2002 must be interpreted cautiously as an Acute Physiology and Chronic Health Evaluation (APACHE) score >10 in cardiac surgery patients indicates that a patient is already at high risk for malnutrition. Patients with an NRS-2002 score >3 are defined as at “risk for malnutrition”, and those with an NRS-2002 score ≥5 as “high risk for malnutrition”, which may be too broad and nonspecific for directing aggressive nutrition support. In contrast, the NUTRIC score was demonstrated in five prospective, but non-randomized studies, as a sensitive measure to identify the nutritional risk and to discriminate between patients with high risk for malnutrition and those likely to benefit from aggressive nutrition therapy [21]. Additionally, nutritional assessment by the NUTRIC score identified patients with a low nutrition risk in whom additional nutritional therapy may have neutral or negative effects. The importance of this is evident in the EDEN trial, which compared the effects of early trophic feeds to early full enteral nutrition (EN) [22]. No difference was shown between trophic vs full feeds in terms of ventilator-free days, infections, or 60-day mortality rate. However, this study was performed in a rather young, initially well-nourished, and nutritional-insensitive patient population with a low NUTRIC score [22]. Casaer et al. [23] compared those with inadequate intake (caloric restriction) to early supplemental parental nutrition (PN) with prior glucose loading. Early initiation of PN to supplement insufficient EN was associated with a higher incidence of infections, delayed recovery, and higher health care costs compared with late initiation of PN. Again, this may have been the wrong target population as the majority of study patients had a short ICU stay, suggestive of a low nutrition risk. Furthermore, tight glycemic control and high-dose glucose loading may have negatively affected the results. It is unlikely that patients having cardiac surgery and at low nutrition risk would benefit from aggressive, early PN, therefore enrolment of these patients in a large clinical trial would be nonsensical. An adequate risk assessment is mandatory to first identify high-risk patients for cardiac surgery before scientifically studying nutrition support implementation.

### Further nutrition assessments tools in cardiac surgery

Ultrasound of the quadriceps muscle is an easy-to-use and readily available tool to measure muscle mass and determine changes in muscle and fat tissue [24–26]. Computed tomography (CT) is also a well-established body composition analysis tool, though more expensive, not risk-free, and difficult to access [25, 26]. Recently, the validity of bioelectrical impedance spectroscopy (BIS) that calculates fat-free mass from measurements of body water has shown promising results in determining nutritional reserve in cardiac patients [27]. In that study a preoperative low bioelectrical impedance phase was associated with malnutrition and increased risk of adverse postoperative events [27]. Yet, the high fluid intake may significantly influence the reliability of BIS. Considering clinical practicability, both CT scan and BIS may open the opportunity for preoperative and postoperative nutrition assessment in cardiac surgery patients. In summary, biochemical inflammatory markers are unlikely to be of use. Ultrasound, CT, and BIS may represent promising future tools allowing quantitative assessment of body composition and, therefore, nutritional status.

## Optimal time point for perioperative nutrition therapy in cardiac surgery patients

Determining the ideal time to start feeding in relation to cardiac surgery represents a crucial factor for nutritional support to be effective. Until now, only a few studies have addressed this question. In regards to timing, the following time windows may be of particular relevance:

- Preoperative: at least 2–7 days before surgery
- Early preoperative: ≤24 hours before surgery
- Early postoperative: ≤24 hours after ICU admission
- Postoperative: >24 hours after ICU admission

One challenging aspect facing perioperative nutritional support is the fact that over half the patients having cardiac surgery are admitted as outpatients on the day of surgery, creating a significant challenge to preoperative nutritional risk assessment and timely intervention. If a beneficial role for a preoperative approach is determined, clinicians will need to overcome this challenge and consider an outpatient approach to optimize the nutritional status prior to admission. In the meantime, the best assessment and treatment window for now is immediately after surgery or soon after arrival at the ICU. Due to the limited evidence on the preoperative or early postoperative identification of these patients, *current practice* currently only allows practitioners to initiate the nutrition therapy on an individualized patient-tailored consideration.

### Enteral vs parenteral nutrition in cardiac surgery patients

Considering international guidelines, there is a strong consensus and most experts will report that EN is always preferred to PN. Following cardiac surgery, critically ill patients are frequently on vasopressor treatment because of an inflammatory response syndrome, vasoplegia, and/or postoperative low output syndrome due to myocardial stunning. The need for vasopressor support further results in marked changes in energy expenditure and frequent intolerance to oral feeding, leading to significant energy/protein deficits and increased risk of malnutrition. Although proven safe [28], EN is often thought to be contraindicated and considered as harmful especially in hemodynamically unstable patients on large doses of inotropes and/or vasopressors, leading to a widespread use of PN in clinical practice. Berger et al. were among the first to provide evidence from a small cohort on the feasibility and safety of early nutrition support in patients after cardiac surgery. Using a well-established paracetamol absorption test, the investigators demonstrated that hypocaloric EN was feasible in such patients with altered hemodynamic status [28]. Kahlid et al. demonstrated in a large scale, multi-center, observational study that mechanically ventilated, vasopressor-dependent patients (mixed population) had a significant survival advantage when EN feeding was started within the first 48 hours after ICU admission, compared to those receiving EN feeding later than 48 hours [29]. Furthermore, in a subgroup analysis, they demonstrated that the sickest patients (on multiple vasopressors compared to those on one vasopressor only) had a more pronounced survival advantage. In addition, nutrition support was demonstrated to be feasible in patients with extracorporeal life support systems (ECLS). All patients on ECLS (in venovenous or venoarterial mode) were fed using existing protocols that emphasize early EN in preference over PN or delayed EN [30]. Notably, the use of paralysis and sedation did not affect feeding tolerance regarding the time to reach goal rate, incidence of intolerance in the first 5 days, or time until first observed bowel motion. In contrast, actual guidelines recommend withholding EN nutrition in hemodynamically unstable patients, though this is primarily based on preclinical and observational studies [31]. The rationale behind this is that the hemodynamic failure may compromise the splanchnic blood flow, causing intestinal ischemia [32], which is associated with high mortality [33, 34].

Given the current evidence, vasopressor use *per se* is not a contraindication to EN. In hemodynamically unstable critically ill or cardiac surgery patients, there is at least some evidence that early EN is absorbed and metabolized without any harmful effect on systemic measurements of oxygenation and perfusion and supportive evidence from a large-scale observational study that this translates into an advantage in terms of mortality [28]. Therefore, early EN may be beneficial in patients after initial resuscitation from critical organ failure. Future well-designed studies are needed for an adequate assessment of this important question.

## Counteracting the inflammatory response - the role of key nutrients

Cardiac surgery with myocardial ischemia/reperfusion and use of CPB is known to be associated with deleterious consequences, resulting from the inflammatory response during cardiac surgery. The duration of CPB time correlates with the extent of the inflammatory response. Furthermore, surgical trauma, ischemia/reperfusion, and contact activation with the CPB circuit result in the release of mainly pro-inflammatory markers, reactive oxygen species, and reactive nitrogen species that contribute to the development of organ dysfunction [35]. In this setting, the use of pharmaco-nutrients, which may exert specific effects on metabolism, the inflammatory response, markers of oxidative stress, and immune cell activity, are of considerable interest. The amino acids glutamine and arginine, lipids such as omega-3 fatty acids, micronutrients such as selenium and zinc, or vitamins A, C, D, and E, are examples of such key nutrients. Despite theoretical promise, several large-scale clinical trials involving these nutrients had disappointing results in the general ICU patient population [22, 36–38]. However, in a small randomized trial in 177 patients, Leong et al. demonstrated that perioperative metabolic therapy with coenzyme Q10, magnesium, lipoic acid, omega-3 fatty acids, and selenium was feasible, safe, and associated with improved redox status, reduced myocardial damage, and shorter length of postoperative hospital stay after cardiac surgery [39]. Although by no means generalizable, the results of that study support the hypothesis that key nutrients can mitigate perioperative oxidative stress and improve cardiac surgical outcomes. Similarly, recent results from a non-randomized open-label study indicate a beneficial effect of perioperative sodium selenite supplementation, whereas the supplementation strategy was still insufficient to compensate for a second decrease in selenium levels during the postoperative course. Given these data, a large-scale multi-center trial was recently launched to study the clinical significance of high-dose (2000 μg) perioperative sodium selenite supplementation in patients at high risk after cardiac surgery [40].

In a recent clinical trial, perioperative nutritional therapy was administered in the cardiac surgery ICU in order to increase myocardial and plasma arginine/asymmetric dimethylarginine ratio and other amino acids [41]. The investigators demonstrated an increase in inflammatory cells in cardiac tissue at the start and end of cardiac surgery, whereas perioperative supplementation during surgery did not affect the myocardial inflammatory response [41]. Similarly, Tepaske et al. performed a double-blind, three-arm clinical trial to determine whether the addition of glycine to oral nutrition may improve the patients’ outcomes after cardiac surgery. It was demonstrated that oral immune-enhancing nutrition reduced postoperative complications, whereas the addition of glycine did not result in any additional beneficial effect [42]. Taken together, recent data do not show a clinical relevant benefit after supplementation of arginine or glycine in patients undergoing cardiac surgery.

So far, preliminary results from small phase II trials on fish oil (FO)-containing emulsions have demonstrated that preoperative FO application is a promising strategy to modulate the biological and clinical response to cardiac surgery [43]. Berger et al. demonstrated that perioperative FO infusions significantly decreased biological and clinical signs of inflammation, in a rather low-risk population of cardiac surgery patients, as reflected by a low mean Euroscore (5), which is routinely used for the preoperative risk stratification in cardiac surgery patients. Furthermore, mainly uncomplicated coronary artery bypass surgery was performed [44]. Given these findings, supplementation of FO may be of particular relevance in patients with more complex procedures with more prolonged CPB time and subsequent pronounced inflammatory response. Manzanares et al. recently conducted a systematic review and included 10 randomized controlled trials (RCTs), in which researchers evaluated FO-containing emulsions in PN or EN in the ICU. The researchers found that FO-containing emulsions may significantly reduce the rate of infections. In addition, FO-containing emulsions were associated with clinically important reductions in duration of mechanical ventilation and hospital length of stay [45]. Further research is encouraged and is needed in cardiac surgery patients to clarify the role of FO.

Pharmaco-nutrition offers a promising approach to enhance the body’s defense mechanisms and to attenuate the deleterious effects of SIRS and improve outcomes. This may be of particular relevance for high-risk patients undergoing complex procedures with prolonged CPB duration and an overwhelming release of pro-inflammatory mediators.

## Main open research topics concerning nutrition in patients after cardiac surgery

The few randomized trials of nutrition support in patients undergoing cardiac surgery are limited to small numbers of patients and demonstrate heterogeneous results, so the experts felt unable to give strong recommendations for clinical practice. Nevertheless, six key messages have been identified by the experts, which are thought to be of clinical relevance in the treatment of these patients:

1. Whenever possible, preoperative optimization of the nutritional state should be targeted in the malnourished patient undergoing cardiac surgery. The increasing number of patients with advanced heart failure and planned VAD implant represent a subpopulation that may as well benefit from optimization of the nutritional state. Thus, determination of nutritional risk, preferably using a structured scoring tool, should be part of the patient’s preoperative assessment.
2. To reach maximum benefit, preoperative nutritional therapy should be initiated in malnourished patients after cardiac surgery at least 2–7 days before surgery (e.g., as part of a preoperative evaluation and optimization therapy) [46].
3. Monitoring of nutrition intake should be routinely assessed daily in patients after cardiac surgery during the ICU stay. In particular, on day 3 all patients should be carefully evaluated as to their nutrition risk and effort should be made to achieve at least 80% of their prescribed protein/energy requirements, either by enteral or parental feeding, as soon as possible.
4. Postoperative nutrition support should be initiated early (0–24 hours after surgery) in patients at high nutritional risk with an expected prolonged ICU stay.
5. Attention to refeeding syndrome may be of importance for patients in whom nutrition support is started after a prolonged period of starvation or in patients with preexisting malnutrition, respectively. In those patients, advancement of feeding should be slower, taking 3–4 days to reach goal, and targeting to adapt to both macronutient and micronutrient special needs [31].
6. If initiated early postoperatively within <24 hours after ICU admission, an additional immune-modulating component (e.g., selenium, fish oil) to nutrition may be considered for patients with complex and prolonged surgical procedures, to counteract the overwhelming inflammatory response.

In extension to the need of reliable data, international standardized procedures such as the ESPEN and IASMEN endorsed strategy for Enhanced Recovery After Surgery (ERAS) are warranted to optimize nutrition support in cardiac surgery patients. In view of the heterogeneous standards of perioperative care in these patients and lack of evidence provided by large-scale RCTs, the multi-modal ERAS program for optimal perioperative care may help to reduce surgical stress, maintain physiological functional capacity, and facilitate postoperative recovery by providing the best available evidence [47].

Furthermore the multidisciplinary group identified six important topics for future research:

1. Targeting preoperative optimization of the nutritional state may result in improved postoperative outcome. Structured scoring tools should be validated and implemented as part of preoperative assessment and to monitor the efficacy of nutrition therapy.
2. In identified patients, the feasibility and clinical significance of early-initiated postoperative nutrition support needs to be evaluated.
3. Dose-finding studies for both macronutrients and micronutrients are needed to answer the questions of “how to supplement patients after cardiac surgery” and “with which combination of nutrients”.
4. To counteract the frequently occurring inflammatory response, the clinical significance of an immune-modulating component (e.g., selenium, fish oil) should be evaluated in patients with complex and prolonged surgical procedures.
5. Validated and reliable assessment of energy requirement in patients after cardiac surgery need to be developed.
6. The role trophic EN might play in the hemodynamically stable patient after initial stabilization needs further evaluation.

## Conclusion

Valid and reliable data are urgently needed to improve the so far non-standardized clinical practice of nutrition screening, assessment, and support in patients after cardiac surgery. Although both inflammatory response and postoperative complications are predictable, clinical practice has several restrictions, limiting optimal nutrition therapy. The accurate identification of patients who benefit most from nutritional therapy presents a clinical imperative requiring validation by adequately powered clinical studies.

## Abbreviations

BIS: Bioelectrical impedance spectroscopy
CABG: Coronary artery bypass grafting
CPB: Cardiopulmonary bypass
CRP: C-reactive protein
CT: Computed tomography
ECLS: Extracorporeal life support systems
EN: Enteral nutrition
FO: Fish oil
ICU: Intensive care unit
IL: Interleukin
MNA: Mini Nutritional Assessment
MST: Malnutrition Screening Tool
MUST: Malnutrition Universal Screening Tool
NRS: Nutritional risk score
NT: Nutritional therapy
NUTRIC: Nutrition Risk in the Critically ill
PCT: Procalcitonin
PN: Parenteral nutrition
RCT: randomized controlled trial
RNS: Reactive nitrogen species
ROS: Reactive oxygen species
SGA: Subjective Global Assessment
SIRS: Systemic inflammatory response syndrome
SNAQ: Short Nutritional Assessment Questionnaire
VAD: Ventricular assist device
